# First hematological and biochemical findings in a rehabilitated giant pangolin (*Smutsia gigantea)* from Southern Cameroon

**DOI:** 10.3389/fvets.2026.1870026

**Published:** 2026-08-17

**Authors:** Marine Montblanc, Jessica Harvey-Carroll, Julie Vanassche, Mark Donaldson, Ellen Connelly, Lisa Hywood

**Affiliations:** 1 Tikki Hywood Foundation, Harare, Zimbabwe; 2 Department of Biological and Environmental Sciences University of Gothenburg, Gothenburg, Sweden

**Keywords:** biochemistry, Cameroon, conservation medicine, hematology, *Smutsia gigantea*, wildlife rehabilitation

## Abstract

Giant pangolin (*Smutsia gigantea*) is one of the least studied pangolin species worldwide, with no published hematological and biochemical data available. We report the first blood parameters from a rehabilitated adult male from Campo Ma'an National Park (southern Cameroon). These preliminary descriptive values, obtained from a single individual sampled during the rehabilitation period, are described and discussed in relation to available data from other pangolin species. They provide the first clinical data for this species and highlight their relevance for medical assessment, health monitoring, and conservation management, while underlining the need for further sampling to establish reference values.

## Introduction

Pangolins are thought to be one of the most trafficked mammals in the world, primarily due to the demand for their meat consumption and scales in traditional medicine (1). Among the eight extant species, the giant pangolin (*Smutsia gigantea*) is the largest African pangolin species with a published record of a 42 kg male pangolin (47). This elusive species inhabits rainforests, swamp forests and wooded savannas across Central and West Africa (2).

The giant pangolin is classified as Endangered on the IUCN Red List (48) and faces severe population declines due to poaching and habitat loss, local extinction has been projected within two decades in countries of its range even without further habitat degradation (3). Despite its conservation importance, the species remains poorly studied, with major gaps in basic biological and clinical data. While conservation programs in Africa focus on pangolin rescue, rehabilitation and reintroduction, the absence of such data limits evidence-based veterinary management and disease monitoring.

Blood analysis, including hematological and biochemical parameters, is a fundamental tool in wildlife veterinary medicine, enabling health assessment, detection of pathological processes, and monitoring of physiological conditions (4). This is particularly important in species such as pangolins, that can conceal overt clinical signs until advanced stages of disease (Authors observations). Blood parameters provide insight into organ function, inflammatory processes, metabolic and nutritional status, and reproductive condition (5). Although such data have been reported for some Asian and African species, they are absent for giant pangolin species. Indeed, blood parameters references have been published for Asian species including Sunda (6–8) and Chinese (9, 10) pangolins. In contrast, for African species, hematological and biochemical information remains scarce with only reference data available for Temminck's pangolin (11, 12) and captive-based values described for the white-bellied pangolin (13).

Establishing hematological and biochemical values for *Smutsia gigantea* is essential to support clinical assessments in rescue centers, enable early detection of health threats and physiological disorders, inform rehabilitation strategies, and strengthen long-term health monitoring of both wild and rehabilitated individuals. Here, we present the first descriptive blood values for this species, obtained from a single rehabilitated individual, providing critical data for the conservation of one of the world's most trafficked mammals.

## Materials and methods

### Admission and clinical management

On August 22, 2025, a giant male pangolin (later named “Akiba”) was confiscated from poachers in Campo Ma'an National Park (Cameroon). On August 26, 2025, he was transported 390 km by road to the Tikki Hywood Foundation (THF) for rehabilitation and care.

Upon admission, the animal showed signs consistent with systemic compromise. The pangolin was found to be lethargic, unable to ambulate, and had reduced responsiveness to external stimuli with a notable absence of the defensive rolling response (2). The individual's inability to maintain a curled posture suggested a state of severe muscular weakness. Physical markers included poor body condition, muscle wasting, and reduced skin turgor, suggestive of dehydration. Body weight at admission was 30.3 kg.

Akiba received supportive and intensive care, including assisted feeding and medical treatment. Enteral nutrition was provided once daily via tube-feeding using a formulated diet, with progressive refeeding volume (from 120 ml up to 600 ml). Oral rehydration salts (100 ml) were also administered during the first 4 days post-admission. Tube-feeding procedure was performed under a light inhalation sedation plane (stage II) using Isoflurane (Piramal Critical Care, Telanga, India) at 5% dose delivered in medical oxygen (5 L min^−1^) via a face mask for 90 s. Pre-oxygenation for 5 min (1 L min^−1^) delivered in flow-by was performed prior to sedation to reduce respiratory side effects following isoflurane inhalation such as hypoventilation or apnea (14).

Medical treatment included a single dexamethasone injection (0.2 mg/kg SC) for management of shock (15, 16), antimicrobial therapy with amoxicillin–clavulanic acid (8.75 mg/kg SC, once daily, 5 days) to treat possible underlying bacterial infection regarding his compromised condition, thiamine (5 mg IM, once daily, 5 days) to prevent refeeding syndrome (17) and iron supplementation (0.08 mg/kg PO, once daily, 3 days) given fatigue and pallor suggestive of possible iron-deficiency anemia (18). After one week of care, body weight increased to 31.4 kg and clinical condition significantly improved.

### Sampling procedure and laboratory analyses

Blood sampling was performed on day 11 post-admission, once the animal was deemed clinically recovered and fit for release based on normal behavioral and clinical parameters: bright and alert demeanor, normal thoracic auscultation and respiratory pattern, pink mucous membranes, normal pace gait without imbalance, nocturnal activity, ability to raise body using forelimbs, effective digging behavior, and strong resistance to handling. Blood sampling was conducted as part of the pre-release medical assessment (Memorandum of understanding, 0022) to ensure the absence of major blood parameters abnormalities that could contraindicate release, based on comparisons with available data from other pangolin species.

Blood sampling was performed under light sedation during daily tube-feeding procedure. Complete morphometric measurements were additionally performed according to parameters described by Perera et al. (19) using a flexible measuring tape. Blood was collected in one EDTA tube and one dry tube by a veterinarian from the medial caudal venous complex using a 16-gauge needle and a 5 ml syringe. In addition, two blood smears were made and stained with a fast-acting variation of May-Grünwald Giemsa staining (RAL 555, Cella Vision) and manual leucocyte differential count obtained. Blood samples were kept refrigerated (4 °C) for 12 h before transportation to Prima Laboratoire (Yaoundé) in an insulated transport container (1 h road transportation at approximatively 4 °C) for hematological and biochemical analyses. No visible hemolysis was noted on macroscopic inspection of the serum. Hematology was performed on a XN-330 analyzer (Sysmex, Switzerland), and serum biochemistry was analyzed on a Cobas Integra 400-Plus system (Roche, France).

As part of the routine health assessment performed prior to release, a fecal examination was conducted using saturated saline flotation method.

### Data visualization

Akiba's blood parameters were compared descriptively with available blood values from other pangolin species. As several datasets were available for Chinese, Sunda and Temminck's pangolins, reference values used for interspecific comparison were selected with a hierarchical approach. Priority was first given to studies including clinically healthy individuals, as physiological status is a major determinant of hematological and biochemical parameters. Among these, preference was then given to data obtained from free-ranging animals, as those are more representative of natural conditions than rescued populations. Finally, if multiple studies met these criteria, those with largest sample sizes were prioritized. For Indian pangolins, the only available study involved a clinically compromised individual, this dataset was included for comparative purposes due to the absence of alternative reference values. Similarly for white-bellied pangolins, only a study based on captive individuals was available and included but comparisons should be interpreted cautiously. Reference intervals were used where available (6, 9, 12), otherwise mean ± standard deviation (13) or single-case values (20) were reported.

Comparative visualization of hematological and biochemical parameters across pangolin species was performed using R (v. 4.2.2, (46)). Plots were generated using the *ggplot2* package (21). For each parameter, mean values were represented using points and error bars indicating reference intervals or standard deviation depending on data availability. Amylase values were excluded from biochemical visualization due to scale differences that impaired graphical interpretation.

## Results

**Figure 1:**
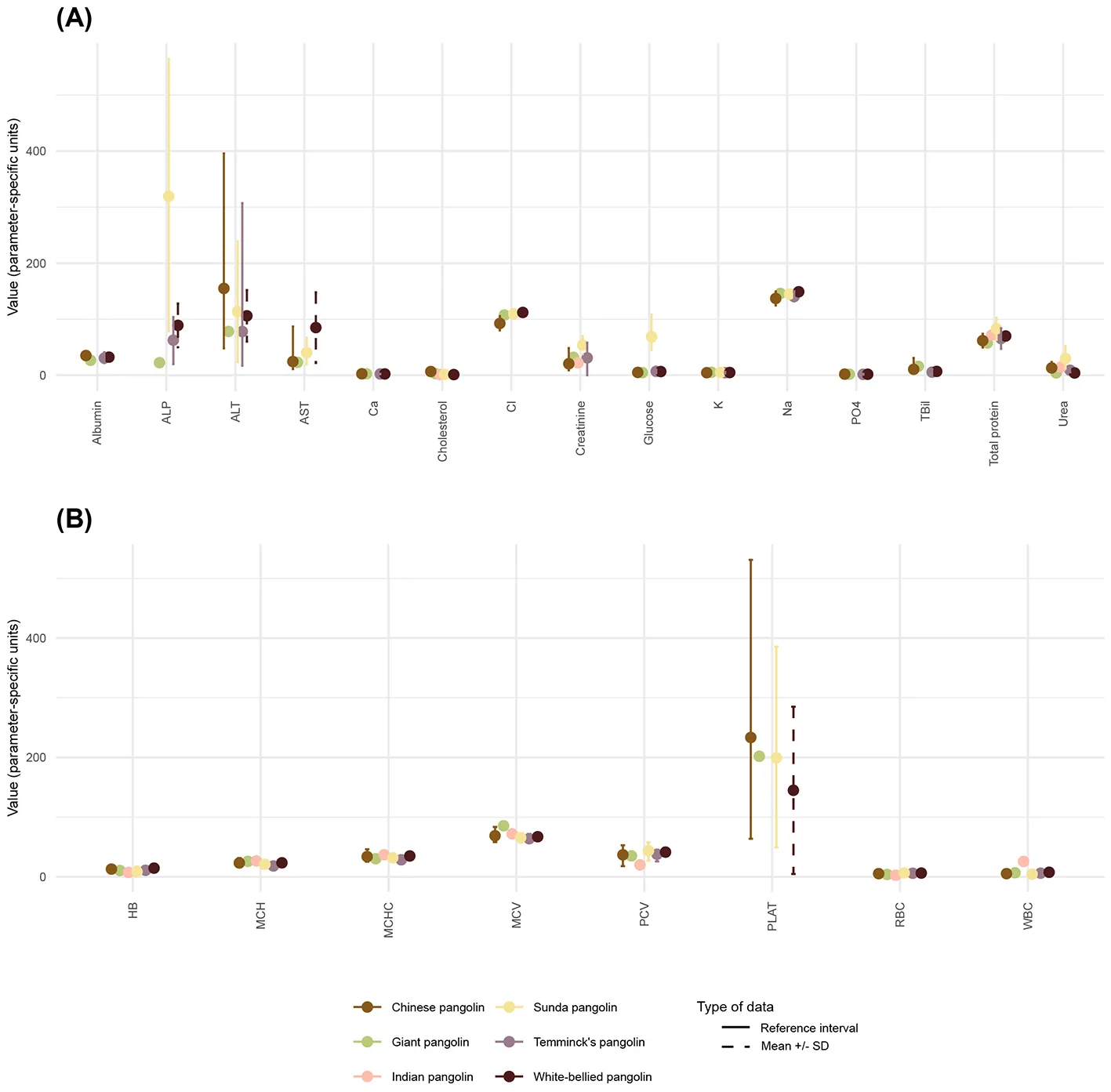
Comparison of biochemical **(A)** and hematological **(B)** parameters across pangolins species. Mean values were used as central estimates. When available, reference intervals are represented (solid lines); otherwise, mean ± standard deviation obtained from literature is displayed (dashed lines). Because reference intervals are not necessarily centered on the mean, error bars may appear asymmetrical. For species represented by a single individual, no error bars are shown.

Hematological and biochemical findings from Akiba are presented in Table 1. Comparison of hematological and biochemical profiles across pangolin species are presented in Figure 1. Akiba morphometric measurements are summarized in Table 2.

**Table 1:** Hematological and biochemical profile of ‘Akiba' compared with published blood values across pangolin species.

Parameter	Akiba (*Smutsia gigantea*)	Temminck's Pangolin (*Smutsia temminckii*) 12	White-bellied Pangolin (*Phataginus tricuspis*)13	Indian Pangolin (*Manis crassicaudata*) *20*	Sunda Pangolin (*Manis javanica*) *6*	Chinese Pangolin (*Manis pentadactyla*) 9
Study population characteristics						
*N*	1	27	17	1	52	100
Sex	M	11 M/16 F	7 M/10 F	F	31 M/21 F	51 M/49 F
Age	Adult	5 Juveniles/22 adults	Adults	Adult	Adults	Adults/subadults
Status	Rehabilitated	Healthy/Rehabilitated	Healthy	Sick	Healthy	Healthy
Origin	Rescued	Rescued (9)/Wild (18)	Captive	Unknown	Rescued	Rescued/wild
Sampling condition	Day 11 post-admission before release; undergone veterinary treatment	Day of capture (free-ranging) or before release (rehabilitated)	Repeated sampling per captive individual	Clinically compromised; died same day	Day 14 post-admission for healthy individuals	Day following capture/rescue; animals with clinical abnormalities excluded
Mass (kg)	31.40	3.40–15.10	—	8.20	>2.00	1.19–6.00
Type of data	Single case	Reference interval	Mean ± SD	Single case	Reference interval	Reference interval
**Hematology**						
Red blood cell profile						
RBC ( × 106/μl)	4.11	3.88–8.31	5.73–7.11 (RI)	2.80	4.02–9.71	2.66–7.73
HB (g/dl)	10.70	7.30–15.00	12.50–16.90	7.50	8.59–17.67	8.18–18.00
PCV (%)	35.20	26.00–45.00	36.00–47.00	20.20	27.30–58.00	18.00–53.00
MCV (fl)	85.64	59.00–72.00	62.90–71.70	72.14	57.30–73.00	58.50–83.59
MCH (pg)	26.03	15.60–21.40	22.20–25.00	26.78	15.40–24.43	18.36–31.61
MCHC (g/dl)	30.40	25.70–32.50	33.40–36.80	37.12	30.04–34.30	25.44–46.32
Platelet profile						
Platelet ( × 10^3^/μl)	202.00	—	5.00–285.00	—	49.30–385.80	63.82–530.93
MPV (fl)	9.00	—	—	—	—	—
White blood cell profile						
WBC ( × 10^3^/μl)	6.90	1.80–10.71	5.14–10.40	25.90	1.23–11.45	2.34–8.58
Neutrophil ( × 10^3^/μl)	2.69	1.19–7.04	1.84–4.48	4.70	0.56–6.21	—
Eosinophil ( × 10^3^/μl)	0.00	0.01–1.95	0.00–0.44	2.80	0.00–0.45	—
Lymphocyte ( × 10^3^/μl)	4.14	0.64–4.35	2.02–6.34	15.30	0.47–2.87	—
Basophil (× 10^3^/μl)	0.00	0.00–0.34	0.00	0.00	0.00–0.25	—
Monocyte ( × 10^3^/μl)	0.07	0.03–1.26	0.05–0.33	3.10	0.00–0.19	—
**Serum Biochemistry**						
Protein profile						
Total protein (g/L)	57.53	47.00–84.00	63.00–77.00	71.70	63.30–102.80	49.50–74.00
Albumin (g/L)	26.70	22.00–41.00	28.20–36.40	—	—	27.00–42.95
Globulin (g/L)	30.83 (Calculated)	21.00–55.00	32.60–42.80	—	—	—
Albumin: Globulin	0.87	—	0.70–1.06	—	—	—
Hepatic function						
ALT (U/L)	78.15	17.00–307.00	60.00–152.00	—	23.13–238.98	48.05–395.83
AST (U/L)	22.91	—	22.00–148.00	—	19.05–67.47	11.00–87.00
ALP (U/L)	22.32	20.00–104.00	50.00–128.00	—	78.64–564.57	-
GGT (U/L)	12.47	—	0.00–9.00	—	—	—
Total Bilirubin (μmol/L)	15.80	1.00–14.00	3.42–10.26	—	—	3.42–30.69
Direct Bilirubin (μmol/L)	2.7	—	1.03	—	—	1.71–10.26
Renal function						
Urea (mmol/L)	4.15	5.30–12.50	2.66–5.33	14.58	12.20–52.22	7.38–23.73
Creatinine (μmol/L)	32.00	0.00–58.00	—	22.10	37.92–69.92 (mean ± SD)	8.84–48.40
Lipid profile						
Cholesterol (g/L)	2.88	—	0.65–1.65	1.14	0.95–1.87 (mean ± SD)	1.97–11.27
HDL (g/L)	0.49	—	—	—	—	—
LDL (g/L)	0.89	—	—	—	—	—
Triglycerides (mmol/L)	0.19	—	—	—	—	—
Electrolytes and minerals						
Sodium (mmol/L)	146.00	132.00–150.00	146.00–152.00	—	141.57–148.15 (mean ± SD)	124.66–149.42
Chloride (mmol/L)	107.4	—	108.70–115.30	—	107.01–112.25 (mean ± SD)	80.05–105.43
Potassium (mmol/L)	5.30	3.10–5.90	4.10–5.50	—	4.75–5.47 (mean ± SD)	3.41–6.64
Calcium (mmol/L)	2.53	2.00–2.60	2.04–2.50	—	—	1.96–3.10
Ionized Calcium (mmol/L)	1.27	—	0.00–2.60	—	—	—
Magnesium (mmol/L)	0.82	—	0.70–0.86	—	—	—
Phosphate (mmol/L)	1.82	1.00–2.60	1.42–2.06	—	—	1.18–2.84
Iron (μmol/L)	17.84	—	—	—	—	—
Other parameters						
Glucose (mmol/L)	4.70	3.60–10.10	5.44–7.77	—	2.40–5.96	2.30–8.63
Amylase (U/L)	1,492.00	267.00–1,533.00	—	—	128.80–969.20	50.50–475.00
Cortisol (nmol/L)	160.4	—	—	—	—	—

Hematology data obtained using a Sysmex XN-330 hematology analyzer and 200-cell manual leukocyte differential counts; serum biochemistry measured on a Cobas Integra 400-Plus system.

RBC, red blood cell count; HB, hemoglobin; PCV, packed cell volume; MCV, mean corpuscular volume; MCH, mean corpuscular hemoglobin; MCHC, mean corpuscular hemoglobin concentration; MPV, mean platelet volume; WBC, white blood cell count; ALT, alanine aminotransferase; AST, aspartate aminotransferase; ALP, alkaline phosphatase; GGT, gamma-glutamyl transferase. “—” = data not available.

**Table 2:** Morphometrics measurements of “Akiba” using Perera et al. (19) morphometric data collection parameters.

Body weight (BW)	31.3 kg					
Snout length (SL)	9.7					
Flesh part of nose (FN)	3.7					
Ear length (EL)	L: 3.1			R: 3.2		
Head length (HL)	21.4					
Neck circumference (NC)	29.5					
Forelimb length (FLL)	29.5					
Shoulder circumference (SC)	77.0					
Body circumference (BC)	83.0					
Head and body length (HBL)	Ventral: 65.0					
Hindlimb length (HLL)	25.6					
Hind foot diameter (HFD)	L: 9.8 length/7.6 width			R: 10 length/7.4 width		
Total body length (TBL)	168.5					
Total diameter in ball (TDB)	151.0					
Tail length (TL)	Ventral: 67.0/Dorsal: 83.4					
Tail base girth (TG)	51.0					
Tail base width (TW)	19.0					
Forelimb claws curvature length (FCL)	L	2nd: 6.6		3rd: 9.6	4th: 7.5	
	R	2nd: 6.6		3rd: 9.4	4th: 7.4	
Hindlimb claws curvature length (HCL)	L	1st: 2.3	2nd: 3.8	3rd: 4.0	4th: 3.0	5th: 1.8

Head and body length was performed ventrally from tip of the nose to base of the anus. Additional measurements include total diameter when rolled into a ball, flesh part of the nose (from last scale to tip of the nose), ventral tail measurement (from base of the anus to tip of the tail), tail base girth and tail base width (ventral measurement). Reported in cm. L, left; R, right.

Blood smear evaluation did not reveal any abnormalities (Supplementary Figure 1). Manual differential leukocyte counts revealed lymphocytes (60%) as dominant white blood cell type followed by neutrophils (39%), and monocytes (1%). Basophils and eosinophils could not be observed on the blood smears. Erythrocytes were round, anucleated, pink, biconcave disks, consistent with pictures from previous pangolin description (12, 20) with no marked anisocytosis, poikilocytosis, hemoparasites, or other erythrocyte abnormalities. Neutrophils were characterized by segmented nuclei and pale cytoplasm containing fine cytoplasmic granules. Lymphocytes were pre-dominantly small, round cells with dense chromatin and a narrow rim of more basophilic cytoplasm. Occasional larger reactive lymphocytes with increased cytoplasmic volume were observed. Monocytes were larger cells with variable nuclear morphology and pale cytoplasm, occasionally containing vacuoles.

Fecal examination revealed presence of strongyle-type eggs and coccidian oocysts (>20 eggs/ooocysts per field at lowest magnification; Supplementary Figure 2). These findings were recorded as part of the standard veterinary evaluation of the individual.

## Discussion

We report the first publication of hematological and biochemical data for endangered *Smutsia gigantea* from an adult male rehabilitated at the Tikki Hywood Foundation, Cameroon. These results should be interpreted cautiously as preliminary descriptive values derived from a single rehabilitated individual sampled after 11 days of intensive care, and should not be considered baseline values for the species.

The absence of prior blood data from giant pangolins likely reflects both ecological and logistical constraints. The species display limited distribution in African tropical forest with peculiar nocturnal and solitary ecology (2), restraining field-based research. In addition, accessing the medial caudal vein in a clinically healthy individual without sedation is technically demanding due to the defensive behavior to roll into a ball, combined with the challenges associated with restraining and manipulating a large-size animal. In remote field conditions, maintaining appropriate sample preservation without refrigeration can also compromise biochemical integrity (22). Moreover, access to diagnostic laboratories equipped and willing to process wildlife samples remains limited in range countries. These constraints may explain the current gap in the literature but also highlight the value of this first documented case.

The number of biochemical parameters evaluated in our study exceed those reported in previous pangolin studies, notably including an extended electrolyte panel (Magnesium, Calcium, Phosphate, Iron), liver function markers (AST, ALT, ALP, GGT), as well as cholesterol and cortisol measurement which provide insights into metabolic, hepatic, and physiological status. Blood gas analysis and lactates measurement were not performed due to the inability to conduct immediate measurements under field conditions. Additional parameters described from other pangolin species such as creatine kinase (CK), and lipase could also have provided further information on muscle activity, and pancreatic function. While CK is a highly conserved enzyme that can be measured across species (23, 24), lipase results are more method-dependent and usually require species-specific immunological assays (25), which may lack reliability in non-model species such as pangolins.

Most of the parameters evaluated in our giant pangolin individual are included in previous reference intervals described in other pangolin species. Packed cell volume (35.2%), red blood cell count (4.11 × 106/μl) and hemoglobin (10.70 g/dl) were within the range described for Temminck's, Sunda and Chinese pangolins. Akiba's RBC indices, however, were characterized by elevated mean corpuscular volume (MCV), high range mean corpuscular hemoglobin (MCH) and low range mean corpuscular hemoglobin concentration (MCHC). Several non-exclusive explanations could result in this pattern. In regard to Akiba's clinical history, this pattern may reflect recovery from previous physiological stress or anemia, as regenerative erythropoiesis is characterized by the release of larger immature erythrocytes containing proportionally less concentrated hemoglobin (26). However, this interpretation could not be confirmed because reticulocyte counts were not performed and serial hematological measurements were unavailable. Alternative explanations include nutritional deficiencies, particularly cobalamin or folate deficiency (27), or species-specific erythrocyte characteristics, as erythrocyte size varies among mammals and may differ between species (28). These hypotheses would require confirmation through additional sampling of healthy giant pangolins and investigation of reticulocyte counts, vitamin B12 and folate concentrations.

The leucocyte profile of Akiba revealed a lymphocyte predominance profile (lymphocyte/neutrophil ratio 0.65). Documented Neutrophil-to-Lymphocyte Ratio (NLR) in pangolins show no consistent pattern when compared across species, with marked variability observed. Lymphocyte dominant profiles have been described in *ex-situ* captive white-bellied pangolins (13) and in a sick Indian pangolin (20), whereas neutrophil-dominant ranges were reported with higher upper reference value in Temminck's, Chinese and Sunda pangolin species. NLR is widely used in clinical practice as a biomarker of systemic inflammation, elevated NLR is associated with increased inflammatory responses and various health conditions (29). The extreme WBC and lymphocyte values reported in the Indian pangolin likely reflect pathological changes rather than physiological variation. Similarly, the higher lymphocyte range observed in white-bellied pangolins should be compared with further study on free-ranging individuals to remove captivity bias limiting the interpretation. Whether Akiba's lymphocyte-dominant leukogram reflects an individual, transient response (e.g., to stress, recent infection or recovery) or a broader species pattern cannot be determined from a single case and warrant further studies. Despite the fecal detection of strongyle-type eggs and coccidian oocysts, no corresponding eosinophilia, basophilia or other hematological abnormality was observed; a mild, early-stage or host-specific subclinical parasitic effect on the blood profile cannot be excluded on the basis of a single sample, and this should be considered when interpreting the leukogram.

Automated leukocyte differential results were not used for interpretation, given the absence of pangolin-specific analyzer validation and a discrepancy between analyzer-reported basophilia and blood smear findings. Validation studies evaluating Sysmex XN-V analyzers in dogs and cats have demonstrated good analytical precision for most hematological variables, but some leucocytes subpopulations such as eosinophils, and monocytes still require manual smear confirmation (30). No validation exists for the Sysmex XN-330 in pangolins, further validation should be conducted to evaluate the analyzer reliability for pangolin species. Differential leukocyte counts obtained from automated analyzers may be unreliable in non-model wildlife species because of differences in cell morphology. Consequently, manual smear evaluation, following the approach used for Temminck's pangolin (12), was therefore considered the most reliable method. Leukocyte morphology appeared comparable to previous descriptions in other pangolin species despite data scarcity in the literature (12, 20), with no abnormalities noted.

Serum biochemical results revealed lower range urea and alkaline phosphatase (ALP) values than those reported for other pangolin species. The most parsimonious explanation is the recent nutritional history of this individual, including fasting prior to rescue followed by artificial refeeding. Reduced protein intake is commonly associated with lower urea concentrations (31), while ALP activity may also be influenced by nutritional status, including protein and micronutrient intake (32). Achieving a nutritionally complete artificial diet, particularly adequate protein intake, remains challenging in myrmecophagous species (33), and these findings are therefore not necessarily indicative of pathology in the rehabilitation context (34, 35). An alternative, untested hypothesis is that species-specific dietary and digestive adaptations may contribute to differences in ALP activity. Giant pangolins preferentially consume larger termite and ant prey than other African pangolin species (36), which differ in macronutrient content (37). However, whether these ecological differences influence biochemical profiles and enzymatic activity cannot be assessed from a single rehabilitated individual and will require investigation in free-ranging individuals.

Conversely, amylase activity was in higher range values for Akiba than reported for other pangolin species. Amylase activity is typically elevated in animals with carbohydrate-rich diets which contrast with protein- and lipid-based diets of myrmeco-termitophagous mammals (37, 38). In our case, increased amylase may reflect the nutritional content of the artificial rehabilitation diet. This hypothesis is further reinforced by previous observations of higher amylase activity in rehabilitated compared to free-ranging Temminck's pangolin (12), suggesting an effect of rehabilitation on this parameter. Pancreatic, intestinal or salivary causes of hyperamylasemia (39, 40) remain alternative explanations but were not supported by any clinical sign. In the absence of species-specific reference limits, it remains pre-mature to conclude hyperamylasemia in Akiba.

Serum cortisol concentration was 160.4 nmol/L. No pangolin-specific intervals currently exist for comparison. In captive Sunda pangolins, mean serum cortisol concentrations of 643.47 ± 457.31 nmol/L have been reported, with higher values in adults (849.65 ± 4 nmol/L) than in subadults (562.2 ± 10 nmol/L), whereas captive Chinese pangolins showed lower adult concentrations (106.2 ± 15 nmol/L) (41). Furthermore, fecal cortisol concentrations were previously reported to be significantly lower in Sunda pangolins rescued from the wildlife trade than in captive-born individuals (42). Direct comparison with the present result in Akiba should, however, be made cautiously because the value reported represents a single time-point measurement obtained under light isoflurane sedation during routine veterinary handling of an individual recovering from recent capture and rehabilitation. Consequently, it cannot be interpreted as representing either resting physiological concentrations or stress-associated cortisol responses. In addition, prior medical treatment, including a single dexamethasone injection administered 11 days before sampling, may have influenced hypothalamic-pituitary-adrenal axis activity. The cortisol value in our study is therefore reported primarily for descriptive purposes. Additional measurements from healthy and rehabilitated giant pangolins will be required to characterize normal physiological variation and evaluate the usefulness of cortisol as a biomarker of stress and welfare in this species.

Comparisons of our data with other pangolin species must be interpreted with caution due to differences in study design, sample size, physiological status, and origin of individuals. Additionally, heterogeneity in data reporting (reference intervals, mean ± SD, or single-case values) further limits direct comparisons. Interpretation of the present hematological and biochemical findings must also account for pre-analytical and analytical sources of variation including age, sex, body mass, circadian and seasonal timing, fasting status, medication, anesthesia, physical exercise, concurrent infection and sampling/storage/transport conditions (43). Importantly, analyses were performed using human-calibrated automated analyzers not validated for pangolins as to date, there are no previous validations, likely due to material limitation (12). This may introduce measurement bias, particularly for indices dependent on cell morphology (44), and for enzyme measurements sensitive to species-specific structural variation. Indeed, differences in isoenzyme structure, co-factor affinity and protein interactions in non-model species may lead to inaccurate activity measurements if species-specific validation is not performed (45). Until proper validation studies are conducted, these data should therefore be interpreted cautiously.

The main limitation of this study is the reliance on a single rehabilitated individual. Therefore, the results presented should be considered as a preliminary clinical description rather than reference data, and interpreted accordingly until validated by further sampling across individuals, sexes, seasons and physiological states. Observed differences between pangolin species may reflect genuine ecological and physiological adaptations, but could also arise from nutritional and rehabilitation context, health status and analytical bias. Despite these limitations, we believe the value of data presented in this manuscript may aid future veterinary treatment of giant pangolins. This report represents an initial contribution toward a more comprehensive comparative clinical framework across Pholidota for which hematological and biochemical information remains critically limited. Notably, comparable data are still unavailable for two further pangolin species, the black-bellied pangolin (*Phataginus tetradactyla*) and the Philippine pangolin (*Manis culionensis*). These findings emphasize the importance of opportunistic data collection in rare and threatened species.

## Conclusion

This study provides the first published hematological and biochemical data for *Smutsia gigantea* from a single rehabilitated adult male. These findings should be regarded as a preliminary description, and extrapolation beyond this individual is not warranted. Larger studies including both sexes, multiple age classes and physiological states will be required to establish species-specific reference intervals. Nevertheless, these data provide an initial clinical benchmark to support veterinary assessment of giant pangolins during rescue and rehabilitation. They represent a first step toward establishing a comparative framework for hematological and biochemical parameters across the genus *Smutsia* and other African pangolins, ultimately supporting evidence-based conservation of the endangered giant pangolin (*Smutsia gigantea)*.

## Data Availability

The original contributions presented in the study are included in the article/Supplementary material, further inquiries can be directed to the corresponding authors.
