# Supplementary material for: First hematological and biochemical findings in a rehabilitated giant pangolin (Smutsia gigantea) from Southern Cameroon
Source: Front Vet Sci. 2026 Aug 17;13:1870026. doi: 10.3389/fvets.2026.1870026 (PMC13526542; doi:10.3389/fvets.2026.1870026)
Supplement: Supplementary file 1 [file Image_1.pdf]

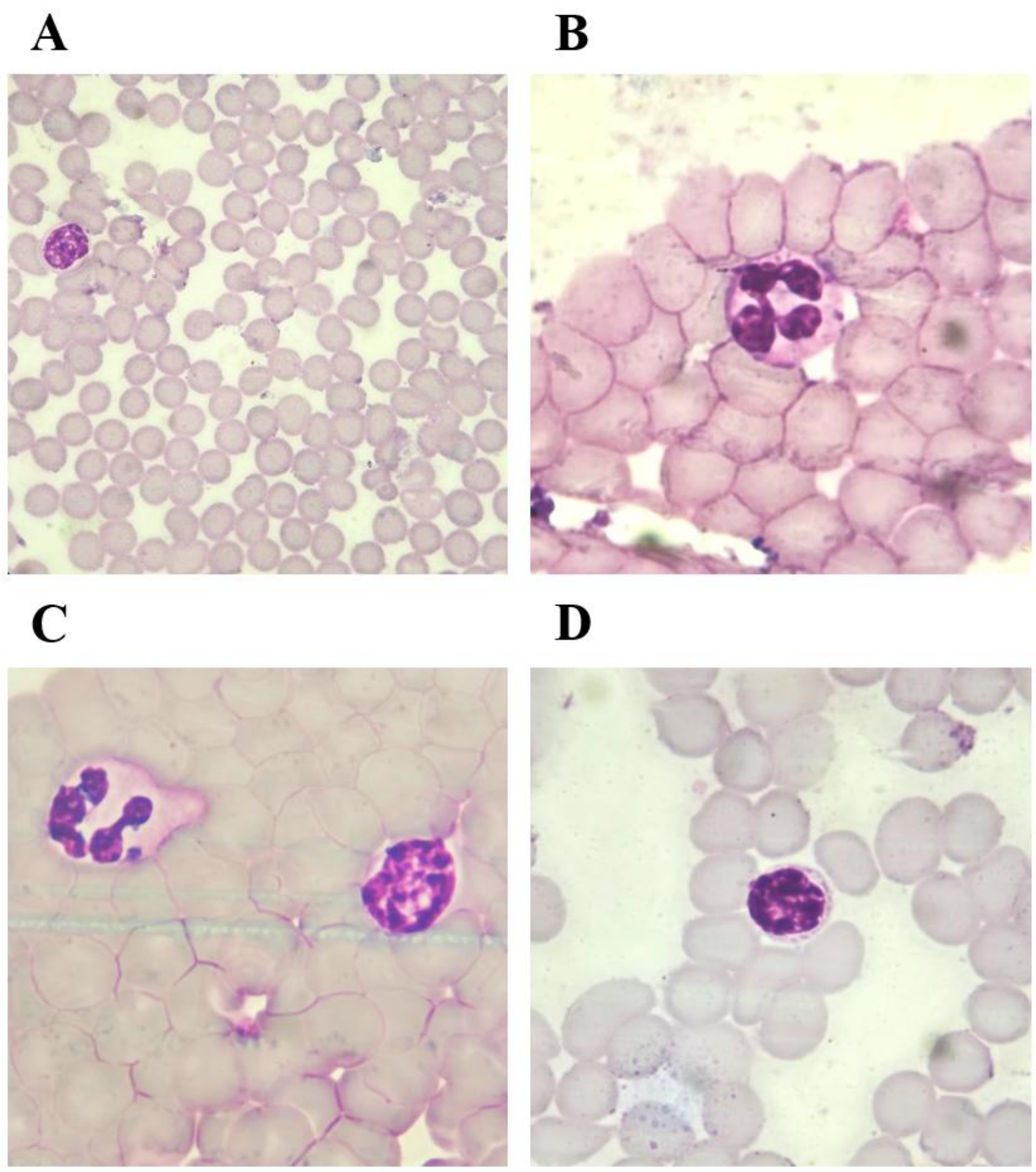

Supplementary Figure 1. Blood smear images from *Smutsia gigantea*. (A) Lymphocyte among red blood cells, (B) Segmented neutrophil, (C) Segmented neutrophil (left) and lymphocyte (right), (D) Lymphocyte. Kit RAL 555 (Cella Vision) staining, × 1,000 objective.
