# Supplementary material for: First hematological and biochemical findings in a rehabilitated giant pangolin (Smutsia gigantea) from Southern Cameroon
Source: Front Vet Sci. 2026 Aug 17;13:1870026. doi: 10.3389/fvets.2026.1870026 (PMC13526542; doi:10.3389/fvets.2026.1870026)
Supplement: Supplementary file 2 [file Image_2.pdf]

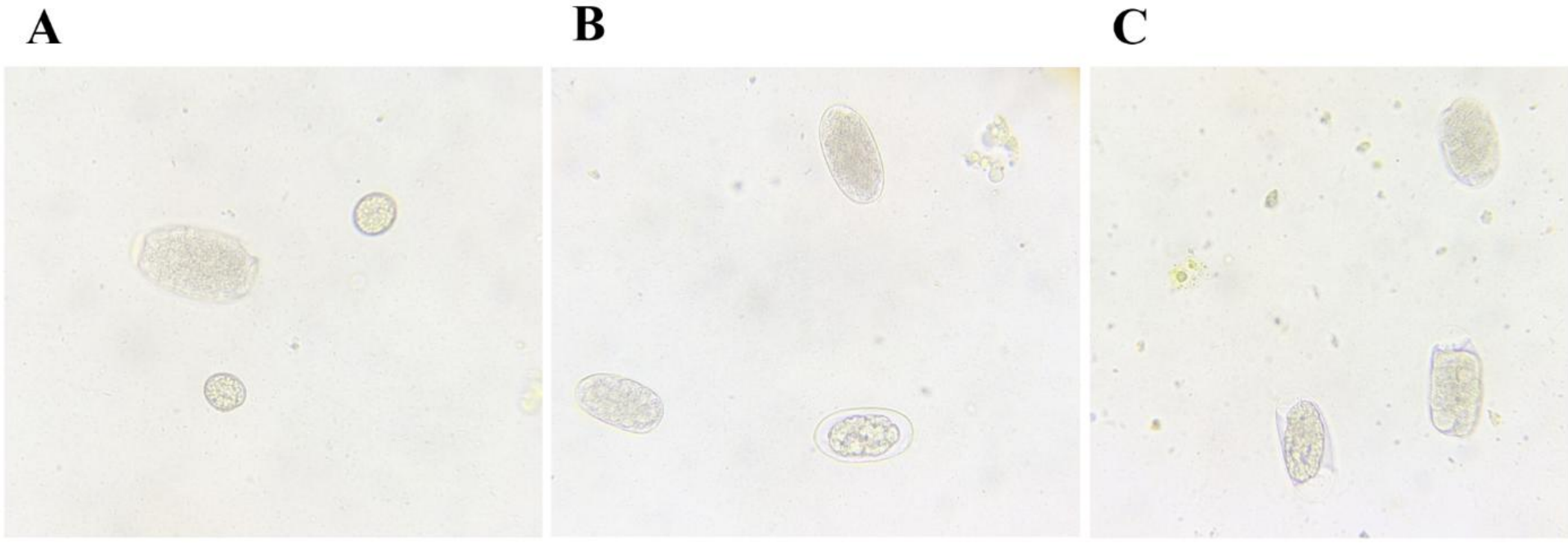

Supplementary Figure 2. Strongyle type eggs and coccidia identified during flotation examination of Akiba's feces. (A) Strongyle type egg and two coccidia × 400 objective, (B) and (C) 3 strongyle type eggs × 400 objective.
